# BMP9 induces postnatal zonal stratification of immature articular cartilage through reconfiguration of the existing collagen framework

**DOI:** 10.3389/fcell.2024.1511908

**Published:** 2025-01-28

**Authors:** Miles Anderson-Watters, Ilyas M. Khan

**Affiliations:** Faculty of Medicine, Health and Life Science, Swansea University, Swansea, United Kingdom

**Keywords:** cartilage, postnatal maturation, collagen, BMP9/GDF2, Benninghoff arcades, zonal stratification

## Abstract

Articular cartilage lines bones in synovial joints, and its main structural element, collagen, has an arcade-like arrangement formed from an initially random network in a process called postnatal maturation. This reshaping of the extracellular matrix is similar across all species and is critical for the lifelong strength and durability of cartilage. Collagen remodelling during maturation is difficult to study because it spans a period of time between birth and puberty, and in larger animals this can be months or years. In this study, we show that growth factor bone morphogenetic protein-9 (BMP9) induces collagen remodelling in intact immature articular cartilage explants within 21 days, generating the characteristic arcade-like structure and zonal anisotropic architecture of adult cartilage. In explants exposed to BMP9, collagen fibrils underwent angular displacement from 19° to 78° with respect to the surface, cell density decreased 1.77-fold, and chondrons were significantly larger. The absence of labelling with anti-COL2¾m, a marker of collagen turnover, showed that the existing fibril network was restructured. We found that stromelysin-1 (metalloproteinase-3, MMP3) gene expression was consistently upregulated, whilst other MMP transcript levels were unchanged or reduced. Remodelling was dependent on proteoglycan turnover and could be inhibited using PD166973. These data suggest a possible mechanism whereby MMP3 induces proteoglycan turnover and depolymerises collagen fibrils enabling them to undergo spatial reorganisation. This process may be driven by tissue swelling, which generates directional strain to align fibrils into an arcade-like pattern. The ability to induce tissue maturation advances the potential for engineering durable and functional cartilage for patients requiring joint repair due to diseases such as osteoarthritis.

## Introduction

Articular cartilage is a soft tissue that provides a smooth, frictionless surface at the ends of bones, allowing pain-free movement and cushioning loads by dissipating forces throughout the skeleton (Sophia Fox et al., 2009). It is made of a dense network of insoluble collagen fibrils that, in newborns, are randomly organised (Rieppo et al., 2009). As animals mature into adulthood, their weight-bearing cartilage adapts to increased joint loading by undergoing a remarkable structural transformation, their collagen fibrils reorganise into an arcade-like pattern (Benninghoff, 1925). This spatial arrangement generates stratification of cartilage into a three-layer zonal architecture: superficial, middle, and deep zones, reflecting the new arrangement of collagen fibrils. This structure is mirrored by the distribution of cells, which are flattened at the surface, round and randomly distributed in the middle zone, and arranged in columns in the deep zone. Chondrocytes express the highly glycosylated proteoglycan, aggrecan (Maroudas, 1976), whose sulphated glycosaminoglycan carbohydrate chains generate a large osmotic pressure gradient that draws water into the extracellular matrix (ECM). The swelling pressure exerted by proteoglycans is restrained in deeper zones by columnar arrays of collagen fibrils, which increase cartilage’s hydrostatic pressure, and this forms the basis of its resistance to dynamic compressive loading (van Turnhout et al., 2011). Postnatal changes in collagen fibril content and organisation directly influence tissue function (van Turnhout et al., 2011; Williamson et al., 2001).

The modification in collagen architecture in articular cartilage occurs as part of a coordinated postnatal maturational process seen in mice (Hughes et al., 2005), rabbits (Hunziker et al., 2007; Julkunen et al., 2010), pigs (Rieppo et al., 2009; Gannon et al., 2015), sheep (van Turnhout et al., 2010), horses (Hyttinen et al., 2009), and humans (Aspden and Hukins, 1981). Sensitive methods that detect collagen turnover in tissues, such as aspartate racemisation (Maroudas et al., 1992) and nuclear bomb pulse ^14^C data (Heinemeier et al., 2016), strongly suggest that following maturation, collagen in joint cartilage remains fixed in place for the entire life of the individual (Eyre et al., 2006). There is no discernible repair or replacement of the collagen network following osteoarthritic disease, lending credence to the observation that joint cartilage ‘once destroyed, is not repaired’ (Hunter, 1743). Attempts to replace diseased or damaged cartilage with cartilage that approximates the correct zonal architecture using cell- or biomaterial-based techniques have yielded to little or no success. Therefore, understanding how articular cartilage undergoes postnatal maturational changes is critical to advancing our ability to create functional and durable tissue-engineered cartilage implants and, in a wider sense, to add to our knowledge of how collagen is patterned in tissues and organs during growth, development, and disease (Holmes et al., 2018; Musiime et al., 2021; Revell et al., 2021; Orgel et al., 2011).

Collagen remodelling from an isotropic network to one that is anisotropic and exhibiting zonal stratification in many animals occurs sometime between birth and puberty. In larger animals the latter time period can span months or years making studying this phenomenon difficult. As a result, the mechanisms driving changes in collagen orientation remain a matter of conjecture and debate (Hunziker, 2009). Bone morphogenetic protein-9 (BMP9) has diverse and pleiotropic developmental effects on cells and tissues, including chondrocytes and cartilage (Mostafa et al., 2019). Previously, we showed that BMP9 had the capacity to alter the trajectory of fibril growth in isolated chondrocytes grown as pellet cultures (Morgan et al., 2020); whether this property extends to intact cartilage is the subject of this study.

We show that the growth factor BMP9 rapidly induces collagen reorientation and columnar re-ordering of chondrocytes in intact immature bovine articular cartilage. Our analysis of this *in vitro* model of cartilage maturation found no significant collagen turnover, leading us to conclude that changes in fibril orientation are due to the reconfiguration of the existing network. We show that proteoglycan turnover is associated with collagen remodelling and that matrix metalloproteinase-3 (MMP3) expression levels rise whereas tissue inhibitor of metalloproteinase-1/2 expression decrease upon exposure to BMP9. Inhibition of MMP activity stops BMP9-induced collagen remodelling. The inter-relationship between collagen, proteoglycans, and water appears to control postnatal changes in fibril architecture, which is relevant to bioengineering tissues with architectures that specify their structural and functional competence (Revell et al., 2021).

## Results

### BMP9 induces collagen fibril remodelling in immature articular cartilage

**FIGURE 1:**
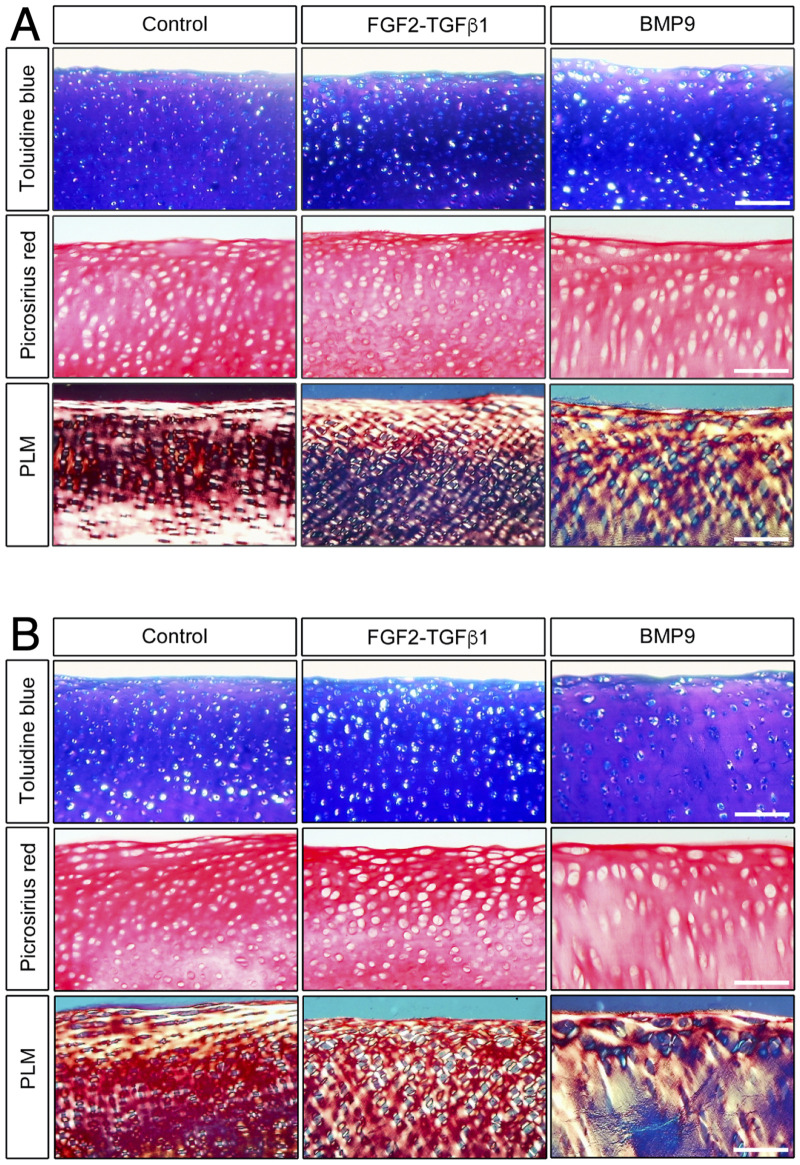
BMP9 induces postnatal maturational collagen remodelling in immature articular cartilage. Immature cartilage explants were cultured in serum-free medium, supplemented with FGF–TGFβ1 (100 ng mL^-1^ and 10 ng mL^-1^) or BMP9 (100 ng mL^-1^) for a total of 21 days. **(A)** Explants were removed from culture at 10.5 days for histological analysis using toluidine blue and picrosirius red staining. PSR sections were also visualised by polarised light microscopy. *Bar = 100 μm*. **(B)** Sectioned and stained explants visualised after 21 days in the culture. Data representative of explants taken from three or more individuals. *Bar = 100 *μm.

When freshly isolated immature articular cartilage chondrocytes are induced to differentiate as high-density pellet cultures in the presence of 100 ngmL^-1^ BMP9, they exhibit collagen architectures that are reminiscent of adult mature articular cartilage (Morgan et al., 2020). Therefore, we tested the ability of BMP9 to induce postnatal maturational changes in intact articular cartilage over a 21-day (d) culture period using 4-mm diameter explants taken from the metacarpophalangeal joints of immature bovine steers. We also included a separate group of explants exposed to fibroblast growth factor-2 (100 ngmL^-1^ FGF2) and transforming growth factor-β1 (10 ngmL^-1^ TGFβ1), which show some key aspects of postnatal maturation of cartilage, such as epiphyseal cartilage resorption, pericellular matrix formation, and accumulation of mature crosslinking, but no interterritorial collagen remodelling (Khan et al., 2011; Khan et al., 2013). Explants were analysed at two timepoints: 10.5 d and 21 d, by histological staining using toluidine blue and picrosirius red to identify proteoglycan and collagen deposition, respectively (Figure 1). In 10.5 d picrosirius red (PSR)-stained sections of control explants, chondrons were flattened at the surface, and in deeper layers, they had an almost uniform size (Figure 1A) (Supplementary Figure S1 *—for comparison with freshly isolated cartilage*). Viewing the latter sections under polarised light microscopy (PLM), the characteristic collagen organisation of immature cartilage was observed; at the surface, the fibrils exhibited a parallel arrangement with strong birefringence, which gradually decreased, indicating a random alignment in the deeper regions. FGF2–TGFβ1 10.5 d-cultured explants were more cellular, and under PLM, there was an increased birefringence signal localised to the pericellular zones of chondrocytes, which was clearer in the mid to deeper regions. In 10.5 d BMP9-cultured explants, PSR-stained sections show a radically different morphology; flattened cells were still apparent at the surface, but there is a distinct increase in the chondron size in the succeeding layers. In deeper zones, chondrons appear elongated and merge to stack upon one another (Figure 1A). PLM imaging of BMP9-cultured explants shows significant changes throughout the tissue depth, particularly in the interterritorial matrix, where increasing birefringence deeper in the tissue signifies greater alignment of fibrils, providing evidence of significant remodelling of collagen. By 21 d, chondrocytes at the surface of control explants have slightly enlarged, but fibrils remain arrayed parallel to the surface with minimal angular displacement, below which there was little or no birefringence, again indicating random alignment of fibrils in deeper regions (Figure 1B). In FGF2–TGFβ1 explants, there was evidence of pericellular matrix remodelling, indicated by the presence of short orthogonal arrays of aligned collagen fibrils surrounding individual chondrocytes from the surface to deeper regions (Figure 1B). In contrast, BMP9-cultured explants under PLM display extensive regions of highly birefringent perpendicularly aligned collagen fibrils with respect to the cartilage surface, a characteristic feature of mature articular cartilage (Figure 1B) (*also* Supplementary Figures S2, 3). Chondron size remained large in the middle zone, and columnar stacking of chondrocytes was evident in the succeeding cell layers (Figure 1B). The other notable features were a reduction in toluidine blue (metachromasia) and PSR staining in BMP9 compared to control and FGF2–TGFβ1-cultured explants, which were more evident after 21 days.

### BMP9-cultured explants adopt the morphological characteristics of mature articular cartilage

**FIGURE 2:**
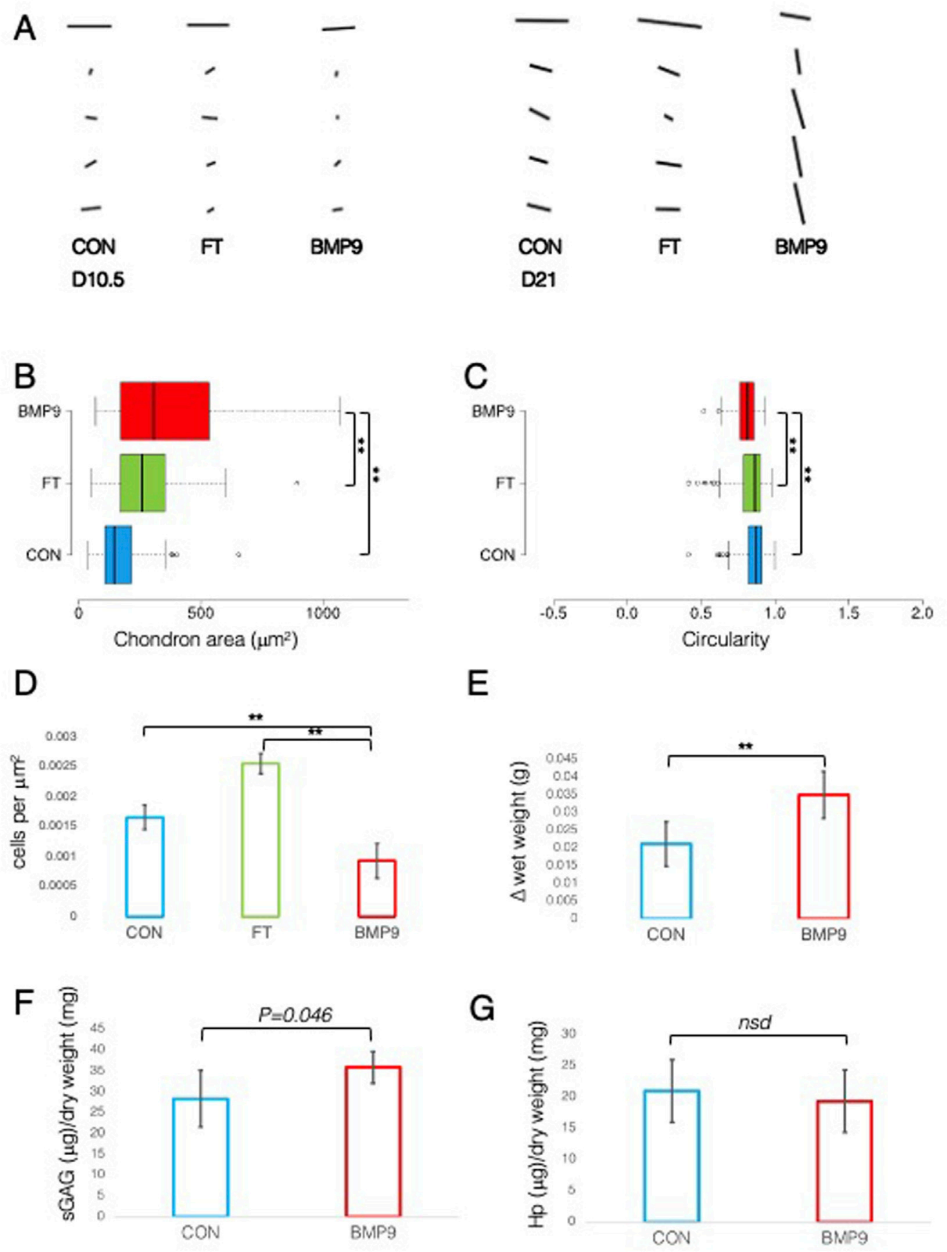
Collagen fibril alignment and cell-specific changes in BMP9-induced postnatal maturation of immature cartilage mirror those observed in adult cartilage. **(A)** FibrilTool collagen alignment analysis of picrosirius red-stained sections from 10.5- and 21-day explants. The length and direction of each bar are equal to the degree of anisotropy of fibrils and the angular displacement with respect to the surface (bars represent sequential sampled areas of 75 μm in height). **(B)** Box and whisker plots of chondron area, and **(C)** circularity index from PSR-stained sections of 21-day cultured explants measured using ImageJ (for both analyses, total cell counts were 113 (control), 162 (FGF2–TGFβ1), and 45 (BMP9), *ANOVA, post Tukey*). Data representative of explants taken from three individuals. **(D)** Histogram displaying the cell density in the surface remodelling zone of explants, expressed as cells per μm^2^ (n = 3, 3 replicates per sample from different surface regions, total cell count per condition was 2411 (control), 3720 (FGF2–TGFβ1), and 1160 (BMP9), *ANOVA, post hoc Tukey*). Mean ± SD. **(E)** Change in wet weight of 21-day control and BMP9-cultured explants (n = 6, *ANOVA, post hoc Tukey*). Mean ± SD. **(F)** sGAG and **(G)** hydroxyproline (Hp) content of 21-day explants as a ratio of their dry weight (n = 6, *ANOVA, post hoc Tukey*). Mean ± SD.

A defining feature of mature articular cartilage are the Benninghoff arcades (Benninghoff, 1925), an arrangement of collagen fibrils in parallel orientation at the surface zone that appears to curve down in a perpendicular direction until they reach the calcified zone of the tissue. We used an ImageJ plugin, FibrilTool (Boudaoud et al., 2014), to provide a quantitative description of the overall direction of fibrils from PLM images of control and FGF2–TGFβ1- and BMP9-cultured explants (Figure 2A). The line length and angular direction for each analysed segment in Figure 2A correspond to the magnitude of anisotropy and the average orientation of fibrils with respect to (wrt) the cartilage surface, respectively. On 10.5 d, fibril alignment is the greatest at the surface zone, where parallel-orientated fibrils predominate. However, it is noteworthy that anisotropy (line length) decreases in BMP9 explants, indicating that a transition is occurring (Figure 2A). Despite the increase in birefringence in the latter sections, the collagen fibrils intersect and cancel each other out, resulting in a smaller measure of anisotropy. After 21 d, the orientation of mid and deep zone collagen fibrils in BMP9-cultured explants is slanted perpendicularly from the surface, whereas in control and FGF2–TGFβ1-cultured explants, anisotropy and angular displacement are only slightly increased (angular displacement for control, 19°; FGF2–TGFβ1, 20°; and BMP9, 78° wrt to the cartilage surface). Another feature of maturing cartilage is an increase in cell size (Decker et al., 2017), which in chondrocytes is demarcated by a thin layer of pericellular matrix forming the chondron (Poole, 1997), the unit cell structure of cartilage. Image analysis of chondron area of picrosirius red-stained sections showed that 21 d BMP9-cultured explants had a significantly larger area (383 ± 271 μm^2^) (*ANOVA, p <* 0.01) than FGF2–TGFβ1 (221 ± 111 μm^2^) or control explants (273 ± 135 μm^2^) (Figure 2B). We analysed the circularity and aspect ratios of chondrons as decreases in the former and increases in the latter are also characteristic of the transition to the mature cartilage phenotype (Figure 2C). Average cell circularity was significantly reduced in 21 d BMP9-cultured explants, 0.78 ± 0.12 (*ANOVA, p <* 0.01), compared to 0.85 ± 0.11 and 0.87 ± 0.09 for FGF2–TGFβ1 and control explants, respectively; a perfect circle equals 1 (Figure 1C). The average aspect ratios of chondrocytes in 21 d BMP9-cultured explants of 1.79 ± 0.66 were significantly increased (*ANOVA, p <* 0.01) compared to FGF2–TGFβ (1.42 ± 0.31) and control (1.46 ± 0.35) explants. The sparsity of cells in articular cartilage is another characteristic property of adult articular cartilage, where there is a greater ratio of extracellular matrix to cell number (Jadin et al., 2005) (Figure 2D). Previous studies culturing immature explants with FGF2–TGFβ1 observed a pronounced proliferative response in chondrocytes (Khan et al., 2011), and this was reproduced in our experiments, where we observed an 1.54-fold increase in cell density (*ANOVA, p <* 0.01) compared to control explants. Cell density was significantly lower in 21 d BMP9-cultured explants by 1.77-fold than in both control and FGF2–TGFβ1-cultured explants *(p <* 0.01). BMP9-treated explants appeared more swollen at the end of the culture period, and this was more pronounced at the surface. The average wet weight of explants was 70.7 ± 0.006 mg for 21 d BMP9-treated explants and 57.2 ± 0.009 mg for control explants (*T-Test, p <* 0.01), representing a 1.23-fold increase on average. The difference between the start and end wet weights for both groups is shown in Figure 2E. Bulk biochemical content analysis of BMP9 explants showed a 1.26-fold increase in sGAG (*T-Test, p =* 0.05) over control explants, but no significant difference in collagen values was observed (Figures 2F, G).

### BMP9-induced collagen fibril reorganisation does not occur through turnover of the existing fibril network

**FIGURE 3:**
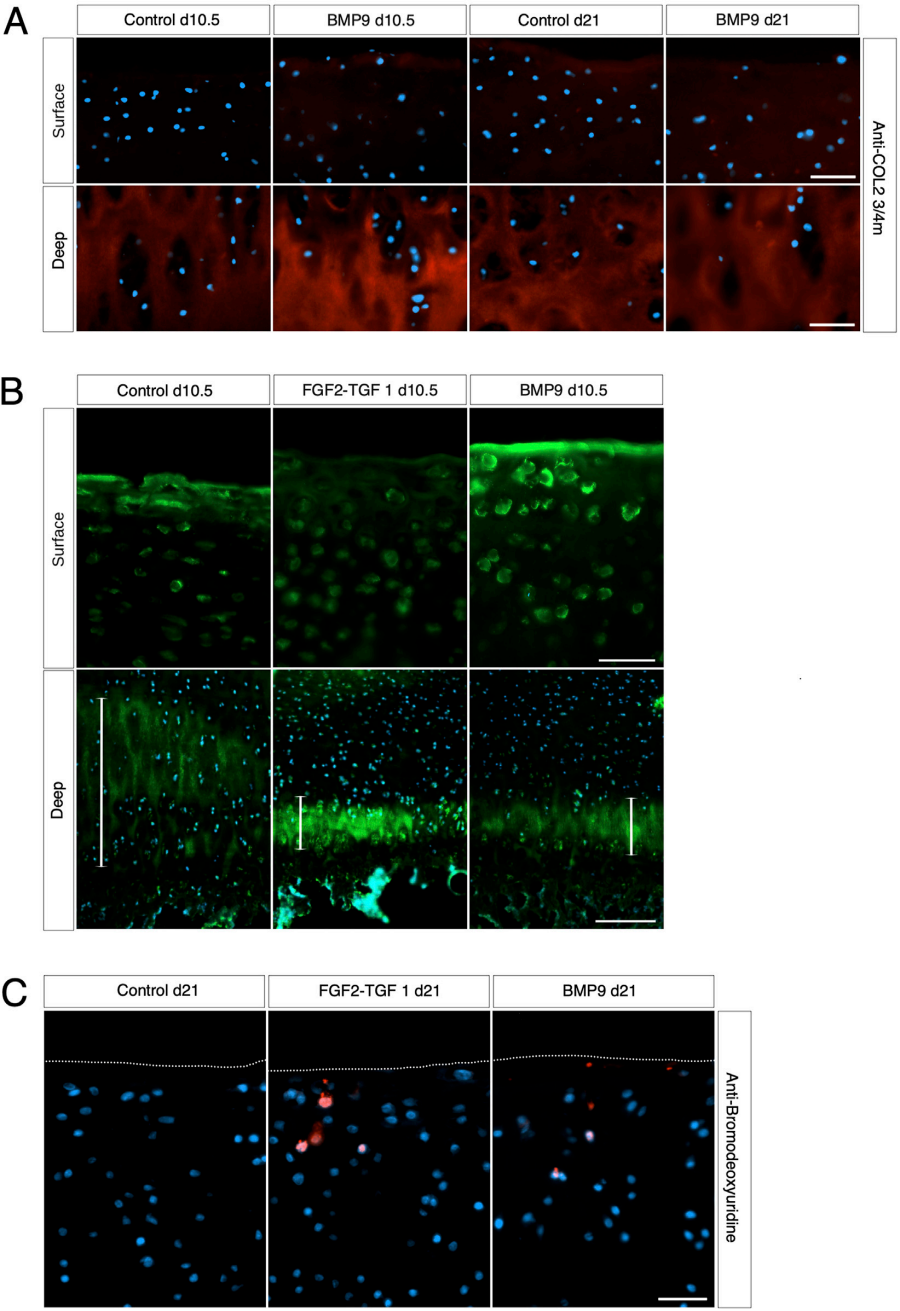
Collagen changes during BMP9-induced remodelling are not the result of fibril turnover. **(A)** Surface and deep zones of control and BMP9 of 10.5-day- and 21-day-treated explants are shown labelled with the antibody anti-COL2¾m. The secondary antibody used was anti-mouse IgG Alexa Fluor 594, and sections were counterstained with DAPI to visualise nuclei. *Bar = 50 μm*. **(B)** DQ gelatin labelling shows the surface (*upper panel*) and deep zones (*lower panel*) of 10.5-day control, FGF2–TGFβ1, and BMP9-cultured explants. The epiphyseal cartilage of the deep label with DQ gelatin in broader bands, and their extent is demarcated with a white line for each explant. *Upper panel bar = 50 μm* and *lower panel bar = 200 μm*. **(C)** BrdU labelling of 21-day-cultured explants. Secondary antibody, anti-mouse IgG Alexa Fluor 594; sections were counterstained with DAPI to visualise nuclei. *Bar = 50 *μm.

**FIGURE 4:**
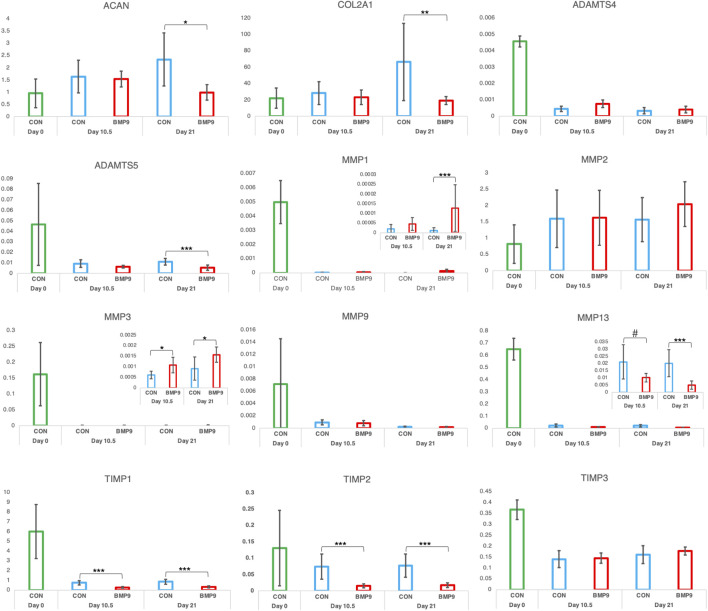
QPCR analysis of absolute gene expression levels of selected biomarkers during BMP9-induced postnatal maturation of immature articular cartilage explants. The *y*-axis of graphs denotes the ratio of transcript levels in ng of the gene of interest divided by the housekeeping in ng. Each panel also includes samples taken from freshly isolated explants (CON, Day 0, n = 3), which were included to provide some perspective on the effect of culture conditions on expression levels. (n = 6, per condition, parametric analysis, *ANOVA, post hoc Tukey*, non-parametric analysis *Kruskal–Wallis, and post hoc Conover/Holm*). # denotes *p = 0.07*. Mean ± SD.

We next examined whether collagen reorganisation in BMP9-cultured explants is due to enzymatic turnover of the existing framework and synthesis of new fibrils with altered orientation. Anti-COL2¾m is a monoclonal antibody that recognises epitopes revealed by collagen denaturation (unwinding) caused by collagenase digestion (Hollander et al., 1994); therefore, the detection of epitopes at 10.5 d in BMP9-cultured explants, when collagen remodelling is highly active, would be indicative of enzymatic breakdown and resynthesis of a new network. Immunofluorescent analysis of antibody labelling of 10.5 d and 21 d BMP9-cultured explants showed no obvious enhancement of labelling in the surface zone, where collagen remodelling is most apparent, compared to that observed in control explants (Figure 3A). Epiphyseal cartilage in the deeper layers of immature articular cartilage is part of the growth plate, which is highly metabolic compared to the overlying hyaline cartilage and provides a natural internal control for collagenase activity. We found high levels of anti-COL2¾m labelling in the epiphyseal zone of all explants, with BMP9 10.5 d explants exhibiting the most intense labelling (Figure 3A). What, then, induces collagen reorganisation in explants? We earlier noted a decrease in toluidine blue staining, signifying proteoglycan depletion in the superficial zone of 10.5 d BMP9-cultured explants despite an overall increase in the bulk content. To confirm the presence of enzymatic activity leading to proteoglycan loss, we performed *in situ* zymography using a fluorogenic substrate, DQ gelatin, to reveal potential metalloproteinase (MMP) activity within 10.5 d frozen sections (Figure 3B). Explants cultured in FGF2–TGFβ1 were included in this assay as studies have shown that this combination of growth factors highly induces MMP activity in the deep zone of immature articular cartilage, leading to tissue resorption (Khan et al., 2011). Green fluorescent labelling indicative of MMP activity was found not only, as expected, in the deep zone of FGF2–TGFβ1 sections but also at lower levels in the deep zones of both control (wide band) and BMP9 (narrower band)-cultured explants (Figure 3B). In the superficial zone of explants, BMP9-cultured explants displayed intense pericellular and weaker extracellular matrix fluorescent labelling, contrasting with the relative lack of labelling observed in FGF2–TGFβ1 sections and weaker labelling in control sections (Figure 3B). PSR-stained sections of BMP9 explants show the rearrangement of cells into columns (Figure 1B and Supplementary Figure S3), and some studies have deduced that this is due to cellular proliferation (Hunziker et al., 2007; Kozhemyakina et al., 2015). Therefore, we also assayed 21 d explants for cell proliferation. Furthermore, we again used FGF2–TGFβ1-cultured explants for comparison as these growth factors have been shown to induce cellular proliferation in superficial zone cartilage (Figure 3C). Control explants showed no evidence of BrdU incorporation; we, however, observed sporadic BrdU incorporation across the surface of BMP9 explants, but there was no definitive pattern that could account for multiple columns of chondrocytes in the remodelling zone (Figure 3C). QPCR analysis of aggrecan (ACAN) gene expression showed a decrease of 2.36-fold (*ANOVA, p <* 0.05) at 21 d in BMP9-cultured explants compared to control explants, a pattern repeated for collagen type II (COL2A1) expression, where a decrease of 3.44-fold (*KW*, *p <* 0.01) was observed (Figure 4). There were no differences in expression between control and BMP9-cultured explants at the earlier timepoint (10.5 d) for either ACAN or COL2A1.

### MMP3 gene expression is upregulated in BMP9-cultured explants

To identify which MMPs were being activated at the surface zone of 10.5 d BMP9-cultured explants, we performed targeted RT-qPCR of enzymes known to be active in articular cartilage during normal metabolism (Figure 4). For this analysis, we also included 21 d cultured explants. There was no change in the expression of aggrecanase ADAMTS4 at either timepoint, but a 0.49-fold decrease at 21 d in BMP9-treated explants for ADAMTS5 levels (*KW, p <* 0.001). Expression levels of gelatinases MMP2 and MMP9 were not altered over 21 d between explants. We identified a 10.7-fold increase in collagenase MMP1 expression in BMP9 explants (*KW, p <* 0.001); however, MMP1 transcript levels are 156-fold lower than MMP13, and therefore, this increase probably has little impact on collagen digestion. MMP13 expression levels in BMP9 explants decreased by 0.48- (*KW, p =* 0.07) and 0.25-fold (*p <* 0.001) at the two timepoints. The only protease that was consistently upregulated in BMP9 explants was MMP3, 1.78-fold at 10.5 d (*KW, p <* 0.026) and 1.72-fold at 21 d (*p* < 0.001). Tissue inhibitor of MMP-1/2 (TIMP) also showed consistent decreases over the two time periods in BMP9-treated explants compared to control explants (TIMP1 0.36- and 0.38-fold (*KW, p <* 0.001) and TIMP2 0.21- and 0.22-fold (*KW, p <* 0.001)).

### Inhibition of MMP activity by PD166793 prevents collagen reorganisation in immature articular cartilage

**FIGURE 5:**
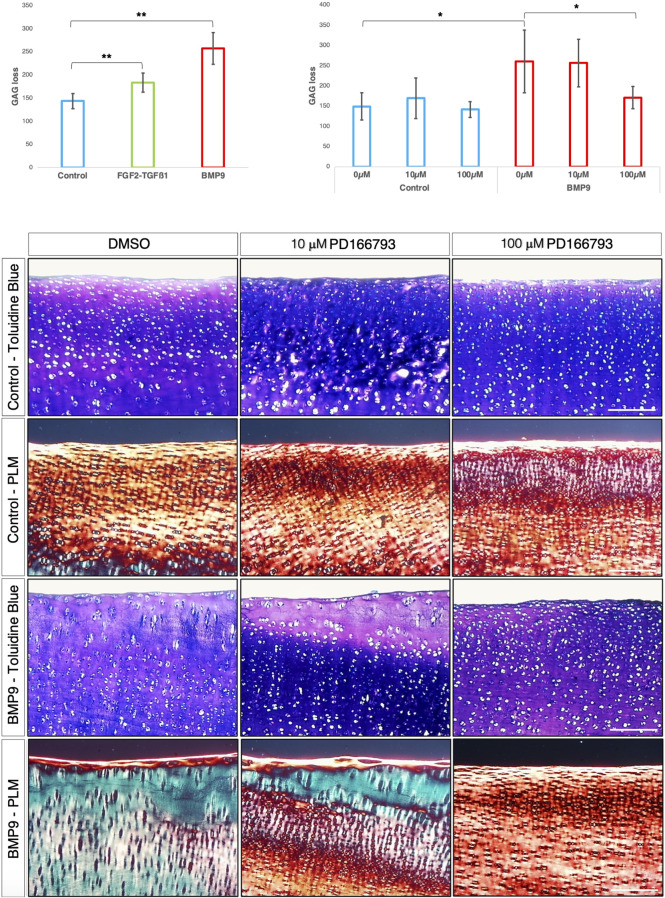
BMP9-induced collagen remodelling is repressed by the MMP inhibitor PD166793. **(A)** Histogram describing the total loss over 21 days of sGAG to the medium from explants (n = 6, *ANOVA, post hoc Tukey*). Mean ± SD. **(B)** Total sGAG loss to the medium over 21 days in control and BMP9 (100 ng mL^-1^)-cultured explants with or without 10 or 100 μM PD166793 (n = 6). Mean ± SD. **(C)** Toluidine blue staining, picrosirius red staining, and polarised light microscopy of control and BMP9-cultured explants cultured for 21* *days with or without the stated concentrations of PD166793. *Bar = 200* μm. Data representative of explants extracted from three different donors. Note the failure of BMP9-cultured explants to partially and completely undergo collagen remodelling when exposed to PD166793; under PLM, collagen fibrils are aligned parallel to the surface, and toluidine staining indicates minimal proteoglycan loss.

MMP3 transcript levels are consistently elevated and TIMP1/2 decreased over the 3-week period in BMP9-cultured explants, and this occurs concurrently with an increase in gelatinase activity at the cartilage surface. Therefore, we hypothesised that it is proteoglycan turnover driven by MMP activity that initiates collagen reorganisation of the existing fibrillar network. First, we analysed proteoglycan loss over the 21 d culture period in cultured explants using the dimethylmethylene blue assay for sulphated glycosaminoglycan (sGAG) and observed a significant increase in sGAG release into the culture medium (*ANOVA, p < 0.01*) for BMP9-cultured explants, which released 1.4- and 1.8-fold more sGAG than either FGF2–TGFβ1 or control explants, respectively (Figure 5A). We then used an MMP3 inhibitor, PD166793 (IC_50_ values are similar for MMPs 2/3/13 but >750 higher for MMPs 1/7/9), to first determine the concentration at which it was able to inhibit sGAG release into the medium of BMP9-cultured explants (this was between 10 and 100 μM; Figure 5B). Next, we cultured immature cartilage explants for 21 d in the presence or absence of 10 or 100 μM PD166793 and BMP9. Histological analysis using toluidine blue staining and PLM of picrosirius-stained sections of control explants showed no overt changes when the culture medium was supplemented with either 10 μM or 100 μM PD166793 (Figure 5C). In BMP9-cultured explants, the loss of proteoglycan in BMP9 was visible through a marked reduction of toluidine blue staining, which is partially counteracted by the presence of 10 μM inhibitor and completely reversed by 100 μM inhibitor (Figure 5C). PLM imaging of corresponding picrosirius red-stained sections shows collagen remodelling, generating perpendicular arrays of fibrils, whose appearance is partially inhibited when explants are co-cultured with 10 μM PD166793 and completely inhibited with 100 μM PD166793.

### BMP9 induces perpendicular alignment of collagen fibrils during pellet culture of immature and mature articular chondrocytes

**FIGURE 6:**
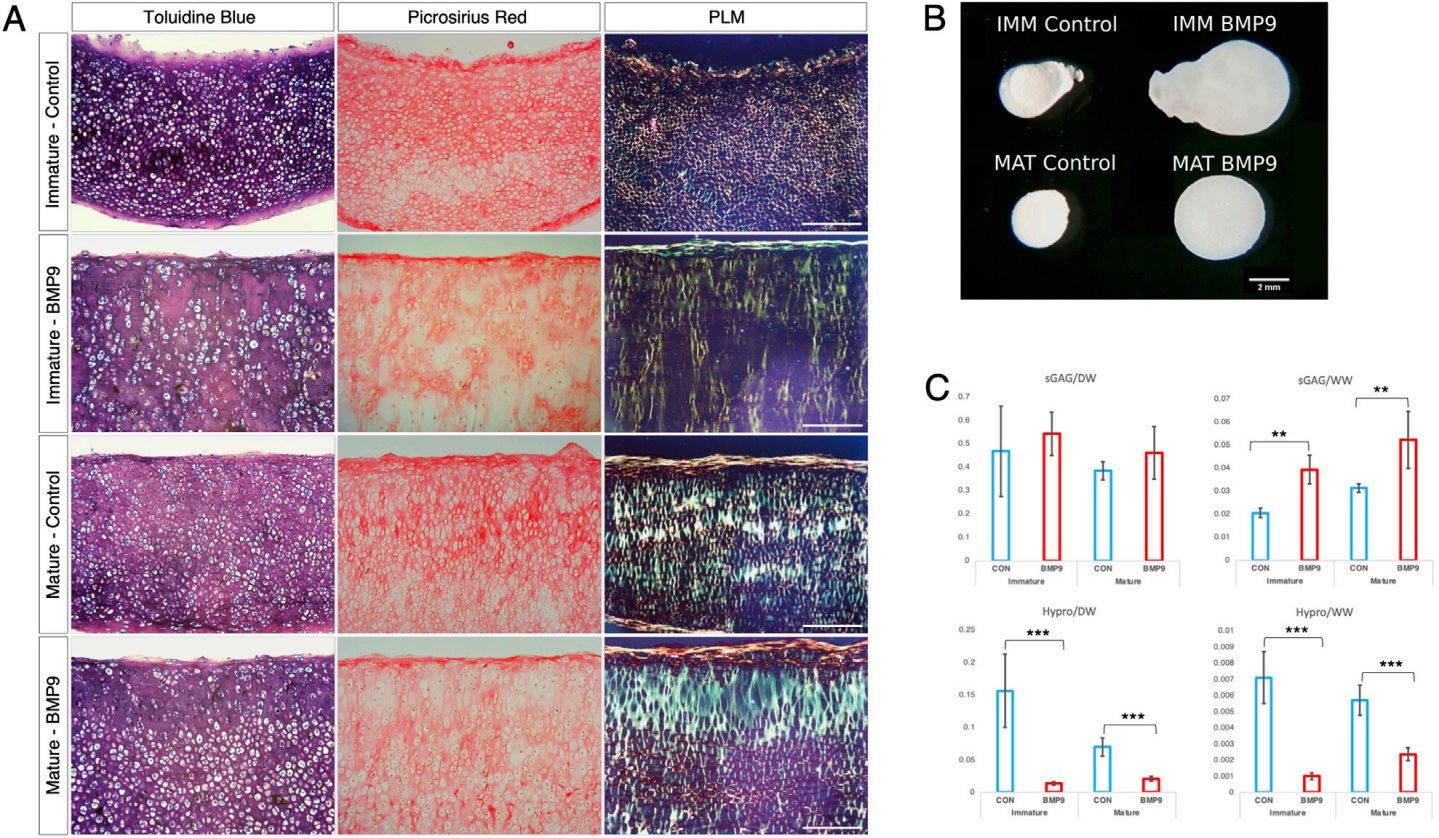
BMP9 induces postmaturational phenotype changes in isolated immature and mature chondrocytes during cartilage growth. **(A)** Toluidine blue staining, picrosirius red staining, and polarised light microscopy of wax sections of 21-day immature and mature chondrocyte pellet cultures. Data are representative of chondrocytes extracted from three different donors. Note the decreased staining for proteoglycan and collagen in BMP9-cultured explants. *Bar = 200 μm*. **(B)** Photographic image of formalin-fixed pellets following 21 days of culture. Bar = 2 mm. **(C)** Biochemical analyses of sGAG and hydroxyproline content (in μg) of pellets expressed as ratios of their dry and wet weights (in μg) (n = 6 per condition, *ANOVA, post hoc Tukey*). Mean ± SD.

A pressing question when engineering articular cartilage is whether chondrocytes have the intrinsic capacity to generate adult articular cartilage, including the correct orientation of collagen fibrils, which are essential for the long-term functional competence of the tissue. To test this, we cultured freshly isolated chondrocytes from both immature and mature articular cartilage as high-density pellets either in the standard chondrogenic medium with or without BMP9 (Figure 6A). Polarised light microscopy shows the radical change in fibril alignment when BMP9 is added to immature chondrocytes, with the orientation of fibrils being predominantly perpendicular to the surface of the pellet, compared to control pellets, where the birefringent signal is predominantly pericellular (Figure 1A). In mature control chondrocyte pellets, birefringent fibrils exhibited increased density in the interterritorial regions of the ECM, and their predominant direction was perpendicular to the surface. In BMP9-treated mature chondrocyte pellets, the interterritorial regions were expanded, forming a band of perpendicularly aligned birefringent collagen fibrils containing elongated chondrons, which in some cases housed multiple chondrocytes (Figure 6A). A notable feature, as with previous experiments with explants (Figures 1A, B), was the reduction in picrosirius red and toluidine blue staining intensity of pellets (Figure 6A). Culture with BMP9 caused a significant increase in volume in immature and mature-derived chondrocytes compared to that observed in control pellets (Figure 6B), and our calculations show that this increase is mainly due to the significant accumulation of water, with an 8.6-fold increase in immature (*ANOVA, p <* 0.0001) and a 3.2-fold increase in mature (*p <* 0.0001) cell pellets. Biochemical analyses of proteoglycan content (sGAG) showed no significant difference in deposition between pellets as a proportion of dry weight (*ANOVA, p =* 0.205) (Figure 6C; however, when normalised to wet weight, values of BMP9-cultured immature, 1.9-fold (*ANOVA, p <* 0.01), and mature, 1.6-fold (*p <* 0.01), chondrocytes were significantly increased (Figure 6C). These data imply that in BMP9-cultured cartilage pellets, the interactions between proteoglycan and water are somehow different; measuring the hydration of mature pellets showed a decreasing trend, 91.8% ± 0.88% *versus* 88.6% ± 0.85% water (*ANOVA, p =* 0.06) between control and BMP9-cultured pellets, mirroring the decrease in the water content of cartilage as it matures. When we measured collagen (hydroxyproline) content in pellets, we found that as a proportion of dry weight, there was an 11.2-fold (*ANOVA, p <* 0.001) and 3.4-fold (*p <* 0.05) decrease in deposition in BMP9-cultured immature and mature pellets compared to their control counterparts (Figure 6C). This difference persisted when the proportion of collagen to wet weight was calculated with a 7.0-fold (*ANOVA, p <* 0.01) and 2.4-fold (*p <* 0.01) decrease in BMP9 pellets (Figure 6C). Therefore, exposure of BMP9 to freshly isolated chondrocytes results in collagen deposition in a perpendicular orientation to the cartilage pellet surface, and when mature cells are employed, there is greater interterritorial alignment of fibrils, implying these differences are developmentally encoded. There are clear similarities between the decrease in picrosirius red and toluidine blue staining in the region of pellets, where significant collagen alignment was observed, and the zone of remodelling in BMP9-treated intact immature cartilage.

## Discussion

BMP9 stimulation of immature articular cartilage can model the postnatal transition of collagen architecture to generate the zonal stratification characteristic of mature adult tissue. We discovered that this transition involves a reconfiguration of the existing collagen network, and a contributory factor is proteoglycan turnover, which, if suppressed using a small molecule MMP inhibitor, stops matrix remodelling. QPCR analysis shows that MMP3 is consistently elevated in BMP9-treated cartilage explants, making it a likely candidate for initiating restructuring of the ECM to the mature configuration. We also show that chondrocytes isolated from immature and mature cartilage are phenotypically distinct when grown as pellet cultures with respect to the matrix they produce, but both generate perpendicularly aligned collagen fibrils characteristic of mature cartilage when stimulated with BMP9. Interestingly, the water content and volume of explants stimulated with BMP9 are significantly greater than controls, and we hypothesise that this is caused by MMP3-induced depolymerisation and hydration of collagen, expanding the tissue and generating a direction of principal strain, which acts to align collagen fibrils perpendicular to the tissue surface.

BMP9 is a member of the TGFβ superfamily of secreted factors and was originally discovered in foetal mouse livers, where it plays a role in tissue growth and homeostasis (Song et al., 1995). It is highly expressed by non-parenchymal cells (Miller et al., 2000) of the liver, where it enters the circulation accounting for 60 percent of serum BMP activity (Scharpfenecker et al., 2007). In serum, BMP9 acts as a vascular quiescence factor inhibiting endothelial cell sprouting through its high affinity receptor ALK1 and co-receptor endoglin (David et al., 2008) but in different contexts supports endothelial proliferation and angiogenesis (Suzuki et al., 2010). Intracellular BMP9 signalling occurs through the phosphorylation of receptor-regulated SMADs 1/5/8, which then form a complex with common mediator SMAD4, translocate to the nucleus where this heteromeric complex modulates gene expression. BMP9 appears to exert diverse and pleiotropic effects during postnatal growth and development of many tissues (Mostafa et al., 2019). In musculoskeletal cells and tissues, it is recognised as a potent osteogenic factor (Varady et al., 2001), stimulating robust and mature bone formation of intramuscularly implanted adenovirus-BMP9-infected osteoblast progenitors (Kang et al., 2004). BMP9 is a potent modulator of chondrogenic differentiation inducing precocious *in vitro* differentiation of articular and auricular chondroprogenitors and stimulating freshly isolated immature chondrocytes to increase matrix synthesis of collagen type II and aggrecan (Morgan et al., 2020; Blunk et al., 2003; Gardner et al., 2023; Hills et al., 2005). The overstimulation of chondrocytes with BMP9 *in vitro* can induce expression of markers of epiphyseal or calcifying cartilage such as alkaline phosphatase and collagen type X (Blunk et al., 2003; van Caam et al., 2015), and it is hypothesised that in ageing tissues, an imbalance in the ratio of ALK5:ALK1 signalling through their respective ligands TGFβ1 and BMP9 promotes osteoarthritic changes in cartilage, an example of antagonistic pleiotropy (van der Kraan, 2014; Mitteldorf, 2019). It also exhibits morphogenetic properties; in pellet cultures of immature chondrocytes, exposure to BMP9 produces cartilage with collagen fibrils aligned perpendicularly to the tissue surface, whereas TGFβ1 induces concentric alignment (Morgan et al., 2020). Yu et al. found BMP9 expression in the interzone of developing mouse joints and, using a model of non-regenerative digit amputation, showed that when the stump is treated sequentially with beads soaked in BMP2 and BMP9 this induces joint regeneration and the formation of a distal skeletal element (Yu et al., 2019). Therefore, as with other tissues, BMP9 has many context-dependent functions that affect cartilage growth and development, significantly including modulation of collagen fibril growth (Morgan et al., 2020), leading us to hypothesise that it induces postnatal collagen network remodelling in intact immature articular cartilage.

In this study, we discovered that BMP9 induces perpendicular reorientation of collagen fibrils in immature cartilage explants within 21 days, although changes in cell shape and collagen fibril order are apparent as early as day 10.5. As remodelling of the ECM proceeds, collagen birefringence under polarised light microscopy increases, highlighting progressive fibril alignment. Simultaneously, the angular displacement of aligned fibrils in BMP9-cultured explants increases from ∼20° in control explants to ∼78°. These latter changes bring about the anisotropic zonal stratification of cells and tissue into distinct superficial, transitional, and radial zones as opposed to a thin surface layer and isotropically arranged cells in the subsequent deeper layer, as found in native immature cartilage (Jadin et al., 2005). Postnatal maturation of cartilage is associated with puberty (Hunziker et al., 2007; Hunziker, 2009), and this starts between months 10 and 12 in bulls, well beyond the post-parturition period (7–28 days) at which time we obtained immature bovine cartilage (Brito et al., 2007). Another quantitative measure of the anisotropy of cartilage upon postnatal maturation is cellular hypertrophy (enlargement, not epiphyseal chondrocyte differentiation) (Hunziker et al., 2007), where we observed an average increase in chondron size of 40% in BMP9-treated explants compared to control explants.

Chondrons are the structural, functional, and metabolic units of cartilage and are composed of one or more chondrocytes surrounded by a thin shell of the pericellular matrix (Poole, 1997). In native maturing cartilage, chondrons in the deep zone become elongated and multicellular, and their aspect ratio (AR, maximum length/width) increases (Youn et al., 2006). This change was also observed in BMP9-treated explants, where the average AR of chondrons increased by 20%. There were only sporadic incidences of incorporation of BrdU in chondrocyte nuclei throughout the cartilage depth of explants. The latter assay was conducted to exclude the possibility that maturational changes in cellular organisation were the result of proliferation, as previously described in rabbit (BrdU) (Hunziker et al., 2007) and mouse cartilage (PRG4 (GFP-CreERt2) cell fate tracking) (Kozhemyakina et al., 2015). Organisational changes in the ECM structure and chondrocyte localisation in cartilage during maturation may be driven by a process of appositional growth. An opposing view, one partially shared by us, is proposed by Decker et al. (2017), who found no significant evidence of appositional or interstitial postnatal cellular proliferation that can account for maturation of cartilage. By using cell tracking of R26-Confetti and GDF5-CreERt2 reporter mice, Decker *et al* found that anisotropic cellular organisation and articular cartilage tissue expansion occurred mainly through cell enlargement and the rearrangement of co-localised cells into columns, similar to integrin β1-dependent columnar chondrocyte formation in the growth plate (Aszodi et al., 2003), where relocated cells remodel the collagen fibril network. It is our contention, which we expand on below, that collagen remodelling precedes topographical cell rearrangement. In our model, we saw no evidence of epiphyseal cartilage resorption and associated tidemark formation, which are also important features of mature anisotropic tissue organisation and result from the formation of the calcified zone, a layer of crenellating tissue that connects the subchondral bone and overlying hyaline cartilage. Thus, BMP9 only induces postnatal maturational changes limited to hyaline cartilage, and this is not unexpected as epiphyseal growth and differentiation are controlled by many well-defined local and systemic regulatory factors (Karimian et al., 2011).

Is the activity of BMP9 in collagen remodelling contingent upon the prior formation of the immature cartilage ECM, or does it induce gene expression programmes that generate mature cartilage phenotypes? We answered this question by growing freshly isolated chondrocytes from immature and mature articular cartilage as pellet cultures in the presence or absence of BMP9. Histological analyses show that chondrocytes from either source are phenotypically distinct, depending on the ECM they generate; however, in both instances, when BMP9 is present, fibrils perpendicular to the surface are produced. The orientation of fibrils in mature chondrocyte pellets was predominantly perpendicular; however, exposure to BMP9 induced the appearance of a band of aligned fibrils in the interterritorial matrix, which became more prominent and formed a distinct lamina across the pellet. The differences between immature and mature pellet formation may be accounted by the fact that prior to and during puberty, joint cartilage is still expanding, whereas following growth plate closure, the matrix is in a process of consolidation. These tissue states are likely governed by distinct transcriptional profiles and biomechanical inputs. These experiments show that fully differentiated, developmentally mature chondrocytes can be used to rapidly tissue-engineer articular cartilage that mirrors the anisotropic structure and function of native tissues.

Therefore, BMP9-induced postnatal maturation of immature articular cartilage provides a tractable model system to investigate the mechanisms driving collagen network remodelling. We first questioned whether changes in fibril order were the result of collagen proteolytic turnover. To address this, we used the antibody anti-COL2¾m, which is capable of sensitively detecting *in situ* collagenase activity (Hollander et al., 1994). Upon digestion with collagenase, the collagen type II triple helix is cleaved between residues Gly^775^ and Gln^779^, producing ¼ and ¾ fragments resulting in helix unwinding and revealing an epitope recognised by the antibody COL2¾m in the latter fragment (Billinghurst et al., 1997). We observed no significant anti-COL2¾m labelling in the surface region of BMP9-treated cartilage, where collagen remodelling was occurring over and above what was present in control explants. Immature hyaline cartilage is continuous in the deeper layers with epiphyseal cartilage, and here, the tissue is dynamically being remodelled, consequently showing significant labelling in the interterritorial matrix with anti-COL2¾m is present at these sites, providing a useful internal control for antibody labelling. A gene expression screen found reduced levels of collagenase MMP13, but an increase in MMP1 was observed. MMP1 transcript levels were, however, extremely low, approximately 156-fold lower than MMP13. Additionally, they were not upregulated above baseline levels at the time (day 10.5) when remodelling was occurring; therefore, MMP1 probably has little or no effect on fibril remodelling. Thus, the absence of detectable collagenase activity strongly suggests that fibril remodelling occurs by restructuring or realignment rather than enzymatic breakdown and synthesis of a *de novo* network. The lack of evidence for appositional growth, the decrease in cell density, and, most significantly, no increase in COL2A1 gene expression over the remodelling timespan are additional supporting evidence that neoformation of the surface zone is a much less likely explanation for the acquisition of anisotropic organisation. Wu et al. (1991) previously suggested that selective proteolysis by stromelysin-1 can initiate remodelling, reorganisation, and growth without having to remove the existing collagen fibril network.

In toluidine blue-stained images of BMP9-treated explants, there was a perceptible decrease in staining and metachromasia in the surface region, indicating proteoglycan loss or turnover, further confirmed by increased labelling by fluorescein-conjugated substrate DQ gelatin, identifying *in situ* activity of MMPs such as gelatinases and stromelysin. We included FGF2–TGFβ1-treated explants in this assay as a useful positive control as a previous study had shown that these growth factors induce elevated levels of MMP activity in the epiphyseal zone, contributing to resorption of the ECM (Khan et al., 2011). A gene expression screen of proteases known to be active in the ECM of articular cartilage revealed a sustained increase in MMP3 (stromelysin-1) over 21 days. MMP3 cleaves aggrecan in its interglobular domain between amino acids Asn^341^ and Phe^342^, releasing the entire glycosaminoglycan-containing portion of this protein (Fosang et al., 1991). Of similar significance is the sustained decrease in expression levels of MMP inhibitory proteins TIMP1 and TIMP2 in BMP9-treated explants, potentially accounting for further increased proteolytic activity. Analysis of DQ gelatin-assayed sections of BMP9-treated explants predicts that if TIMP activity falls in the deep zone, then the fluorescence signal should increase, but the zone of activity decreases compared to control explants, suggesting that reductions in TIMP1/2 expression may facilitate proteoglycan depletion in the upper zones of cartilage. The measurement of sGAG loss into the culture medium also confirmed proteolytic degradation of proteoglycan, and its return to baseline levels of turnover was used to determine the concentration of MMP inhibitor PD166793 to achieve this. The presence of 100 μM PD166793 inhibited collagen remodelling at the surface of BMP9-treated explants, implying that reorganisation of fibrils is conditional upon proteoglycan depletion or turnover.

Fibrillar collagens resist hydrodynamic, compressive, and tensile forces, and the amount and direction of strain positively correlate with the degree of collagen fibril alignment (Hapach et al., 2015). What, then, generates the internal tension within cartilage that aligns fibrils during BMP9 induced remodelling? Water (Cederlund and Aspden, 2022)? The loss of proteoglycan and depolymerisation of collagen not only free collagen to deform but also induce tissue swelling, and we suggest that water ingress generates the directional tensional stress to align fibrils. Proteoglycans within the cartilage ECM contribute to the fixed charge density and osmotic pressure, and this swelling pressure is counteracted by elastic forces within the collagen fibril network (Maroudas, 1976). With proteoglycan turnover, there is a reduction in swelling pressure, and consequently, the collagen matrix becomes less stiff (Maroudas et al., 1985), allowing it greater freedom to deform. The numerous glycosaminoglycan chains of the large aggregating proteoglycan aggrecan form electrostatic interactions with collagen, and this is suggested to occur through the axial d-periodic repeat gap of collagen, leading to the formation of an interlocked matrix exhibiting spatial organisation (Junqueira and Montes, 1983). Proteoglycans assemble with regularity along collagen type I fibrils in orthogonal arrays in tendon (Scott, 1991), and similar ordered associations between collagen type II and chondroitin sulphate containing proteoglycans have been confirmed using electron microscopy (Poole et al., 1982), low angle X-ray diffraction (Ronziere et al., 1985), and colloid force spectroscopy (Rojas et al., 2014). Therefore, proteoglycan turnover also has the effect of removing an imposed supramolecular order, further increasing the freedom of fibrils to be aligned under tension. Explants undergoing collagen remodelling significantly increase their water content during the culture period (∼66%), mainly at the surface region; the cause is two-fold. First, MMP3 activation not only induces proteolytic depletion of cartilage proteoglycans but also collagen type IX. Collagen type IX is a fibril-associated collagen with interrupted helices (FACIT) that is covalently linked to the surface of collagen type II fibrils (van der Rest and Mayne, 1988). Its amino terminal non-collagenous domain (NC4) and adjacent collagenous domain (col3) project away from the fibril surface and are available to interact with other molecules in the ECM (van der Rest and Mayne, 1988). In articular cartilage, collagen type IX NC4 domains stabilise collagen type II interfibrillar interactions through covalent linkages to adjacent type II telopeptides and type IX NC4 domains (Wu et al., 1991; Wu et al., 1992). MMP3 proteolytically cleaves collagen type IX in its C-terminal NC2 domain, releasing NC4 and uncoupling it from the surface of type II fibrils (Wu et al., 1991), which together with its telopeptidase activity causes the depolymerisation of collagen type II fibril networks. Second, prior to the loss of proteoglycan, collagen is not fully hydrated as water molecules are drawn to glycosaminoglycan covalently bound to aggrecan; however, once glycosaminoglycan density is reduced by proteoglycan degradation, depolymerised collagen is free to hydrate. Collagen is highly hygroscopic, and as an extended molecule, it presents many donor and acceptor sites for hydrogen bonding. In its fully hydrated state, it contains three times as much water volumetrically as the collagen ground substance alone, leading to tissue swelling (Andriotis et al., 2018). Andriotis et al. have shown that fully hydrated collagen is subject to fewer intermolecular van der Waals and/or electrostatic forces, resulting in increased intrafibrillar distances. This change opens deformation pathways that were previously closed (Andriotis et al., 2018). There is direct evidence of this mechanism in action in the developmentally regulated selective proteolysis of collagen type IX, which triggers corneal stroma matrix swelling (Fitch et al., 1988). Fitch et al found that when collagen type IX antibody reactivity in the stroma was lost, it coincided with matrix swelling. Type IX protein was found in soluble fractions of extracts, whereas collagen type II remained intact in the stroma and was found in insoluble fractions. Therefore, the ingress of water into the cartilage surface has the potential to hydrate a depolymerised collagen network and imposes a directional strain upon the tissue that is perpendicular to the surface. We hypothesise that the tensile stress directed to the surface as cartilage hydrates is sufficient to radially realign fibrils. If true, then in the absence of further collagen synthesis, the collagen density in this region should decrease as the tissue volume increases, and this should be reflected by a reduction in picrosirius red (collagen) staining, which is what we observed experimentally. The volumetric expansion of tissue, together with increased chondron size (Decker et al., 2017), accounts for the reduction in cellular density. In addition, proteoglycan composition as a fraction of dry weight should decrease as postnatal maturation proceeds as collagen remodelling is dependent on proteoglycan turnover, and when these analyses are performed, significant decreases ranging between 25% and 50% are observed in maturing cartilage (Gannon et al., 2015; Levin et al., 2005; Brama et al., 1999). Finally, collagen type IX has long or short, alternatively spliced isoforms that differ in the presence of the NC4 domain; their selective expression, along with collagen crosslinking lysyl oxidases (Zhang et al., 2017), which may underlie some of the phenotypic differences observed between immature and mature chondrocyte ECM growth during pellet development, as has been shown in the developing avian cornea (Fitch et al., 1995).

Collagen alignment can occur through direct or indirect physiological and non-physiological mechanisms (Revell et al., 2021). In tendons, fibril alignment is along the principal longitudinal axis of tension, and fibrils are assembled directionally from plasma membrane invaginations called fibropositors (Canty et al., 2004). These structures have not been detected in chondrocytes, and this is unsurprising as these cells are surrounded by a thin shell of the pericellular matrix, although chondrocytes are capable of directional polarisation through the orientation of mechanoresponsive primary cilia (Meier-Vismara et al., 1979; Poole et al., 1985). Another reason for disregarding the latter mechanism for remodelling in cartilage is our data strongly suggest an indirect mechanism of fibril alignment. Fibroblasts, using cell traction forces, are capable of long range (1.5 cm) deformation and remodelling of collagen gels to form aligned ligamentous straps between cell masses (Harris et al., 1981). The generation of tractional forces is a product of cell migration, which occurs through the formation of lamellipodia and filopodia over traction sites—features noticeably absent in chondrocytes from intact cartilage (Morales, 2007). Although we have shown bulk loss of proteoglycans, further analyses need to be conducted using antibodies that can detect aggrecan degradation products to precisely map the regions of increased turnover in the explant. Similarly, MMP3 expression localised by *in situ* hybridisation would provide further evidence of its role in collagen realignment.

In conclusion, this study has uncovered a possible mechanism governing collagen remodelling during postnatal articular cartilage maturation that generates Benninghoff arcade-like structures (Benninghoff, 1925). The central idea is that collagen–collagen and collagen–proteoglycan interactions provide spatial order in the ECM, but when enzymatic activity induces proteoglycan turnover and causes collagen depolymerisation, this allows fibrils the freedom to realign. Water hydrates depolymerised collagen fibrils, imparting further freedom whilst simultaneously inducing tissue expansion, which generates tensile stress to align fibrils along the direction of principal strain, in this case, perpendicular to the surface. This hypothesis can be tested in future studies in several ways to induce collagen reorganisation through, for example, specific enzymatic action or inhibiting the process by preventing tissue expansion. Furthermore, it will be instructive to know whether BMP9-treated cartilage is stiffer or whether further biochemical or biomechanical conditioning is required to fully mature the structure. Collagen is the most abundant protein in the human body and serves as the backbone of connective tissues, which form a myriad of specialised structures with tissue-specific three-dimensional organisations, including the cylindrical, calcified concentric lamellae of bone, the capsules that enclose tissues and organs, the circumferentially aligned fibres of arteries and veins, and the orthogonal lamellae of the cornea. Understanding precisely how these structures are made is important; only then can we engineer them to repair, replace, or regenerate tissues when they are damaged, diseased, or absent. The most successful method of replacing diseased cartilage is using donor transplants of pristine cartilage, not an economically viable solution for millions who suffer from osteoarthritis. The results of this study show that engineering durable cartilage implants with a mature anisotropic organisation may be closer to realisation.

## Methods and materials

Articular cartilage explant culture: 7–28-day-old bovine feet were freshly obtained from local abattoirs, thoroughly washed, disinfected with 70% ethanol, and skin removed. Institutional ethical permission was granted for this study as the bovine feet are by-products of the human food chain, and their use complies with the Retained Animal By-Products Regulation (EC)1069/2009. In a sterile hood, metacarpophalangeal joints were opened using a size 23 scalpel, and cartilage explants from the inner aspects of the medial and lateral condyles were cored using a 4-mm biopsy punch. Explants were removed by cutting the inferior surface of the cores. These explants were placed in adjacent pairs or triplets in 24-well culture dishes (Corning, UK), with 1.5 mL of the chondrogenic medium consisting of Dulbecco’s modified Eagle medium (DMEM) high glucose and GlutaMAX™, 1 x insulin–transferrin–selenium (ITS). In addition, 100 μg ml^-1^ L-ascorbic acid 2-phosphate sesquimagnesium salt hydrate, 10 mM HEPES (pH 7.5), and 50 μg mL^-1^ gentamicin were included, and the explants were allowed to equilibrate overnight. For treatments, control explants had their medium replaced, while growth factor additions included bone morphogenetic protein-9/growth and differentiation factor-2 (BMP9/gdf2, Peprotech Ltd., UK) at a concentration of 100 ng mL^-1^, fibroblast growth factor-2 (FGF2, Peprotech Ltd., UK) at 100 ng mL^-1^, and transforming growth factor-β1 (TGFβ1, Peprotech Ltd., UK) at 10 ng mL^-1^. The culture medium was replaced every 4th day for both 10.5- and 21-day cultured explants. For inhibitor studies, PD166793 (Tocris, UK), dissolved in dimethyl sulphoxide (DMSO), was added at a final concentration of either 10 μM or 100 μM to explant medium, with control explants receiving the same volume of DMSO. Inhibitor was replenished at medium changes.

Histological analysis: explants were fixed in 4% paraformaldehyde in phosphate-buffered saline (PBS) overnight at 4°C and then processed into wax, and 8 μm sections were cut. Dewaxed and rehydrated sections were either stained for proteoglycan using the metachromatic stain 1% (aq) toluidine blue for 60 s or collagen by picrosirius red for 60 min following treatment with 1% w/v hyaluronidase for 60 min at 37°C to remove proteoglycan. Polarised light microscopy was conducted using a Leica DMLP Microscope equipped with a circular stage where picrosirius red stained sections were orientated with the surface zone at 45˚ to the crossed polarising filters in order to detect birefringent light signal. Image analysis: the cell density of explants was quantified using ImageJ using fluorescent images of 4′,6-diamidino-2-phenylindole (DAPI)-stained histological sections. The image was converted to 16-bit greyscale and threshold adjusted to highlight cell nuclei, and the merged nuclei were separated using the watershed function and counted using the *Analyse Particles* function. The same technique was used to highlight chondrons in picrosirius red-stained sections and measure their circularity, aspect ratio, and area from spatially calibrated images (approximate dimensions 300 × 100 microns, l x w) using the *Analyse Particles* function. Analysis of collagen fibrils was performed using the ImageJ plugin FibrilTool (Boudaoud et al., 2014), which provides a quantitative description of the average orientation and anisotropy of fibrous arrays in tissues. Five sequential 75 μm × 75 μm areas from the surface of polarised light microscopy-imaged sections of explants were analysed for anisotropy and average orientation, and the outputs from the log file were re-plotted to lines (line length = anisotropy and line direction = average orientation).

Biochemical analysis: explants for cryosectioning or biochemical analysis were snap-frozen in beakers filled with n-hexane pre-cooled in a dry-ice and ethanol bath. Frozen explants were digested in 20 mM sodium phosphate (pH 6.8), 1 mM EDTA, 2 mM dithiothreitol, and 300 μg mL^-1^ papain at 60°C for 60 min. Proteoglycan in explants and explant culture medium was determined indirectly through the measurement of sulphated glycosaminoglycan (sGAG) content in papain-digested solutions using the dimethylmethylene blue assay and shark chondroitin sulphate to generate a standard curve (Farndale et al., 1986). Collagen content was determined indirectly through the quantification of hydroxyproline content of papain-digested solutions by alkali hydrolysis as described by Cissell et al. (2017).

Antibody labelling: cryosections, 5 μm, were cut and washed in Tris-buffered saline/0.1% (v/v) Tween-20 (TBST) to remove optimal cutting temperature cryomountant (OCT: RA Lamb), treated with 1% (w/v) hyaluronidase in TBST for 60 min at 37°C to remove proteoglycan, blocked with 10% goat serum in TBST for 30 min, and then incubated with 1 μg mL^-1^ of the primary antibody, mouse monoclonal IgG anti-COL2¾m in TBST, overnight at 4°C. Following several washes at room temperature with TBST, labelled epitopes were detected using 5 μg mL^-1^ of the secondary antibody, goat anti-mouse Alexa Fluor 594 (Thermo Fisher Scientific, UK) for 60 min, washed in TBST, overlaid with DAPI containing antifade mounting medium (VECTASHIELD, VectorLabs, UK), and imaged using an Axio Imager M1 Fluorescence Microscope (Zeiss, UK). For the *in situ* detection of bromodeoxyuridine (BrdU) incorporation in immature cartilage, 10 μg mL^-1^ BrdU was added to the medium for the final 3 days of cultures of 21-day explants, at the end of which explants were washed in DMEM, fixed in 4% paraformaldehyde in PBS overnight 4°C, and processed to generate 8-μm wax sections. Standard procedures for antibody labelling were performed, as described above, with the addition of an incubation step of 60 min in 0.2 M HCL to denature DNA, followed by incubation with 0.1 M sodium borate buffer (pH 8.5) to neutralise acid. In addition, 1 μg mL^-1^ mouse monoclonal antibody G3G4 (obtained from Development Studies Hybridoma Bank, U. Iowa, United States) was used to detect labelled DNA and goat anti-mouse Alexa Fluor 594 as a secondary antibody.

*In situ* zymography: frozen 5-μm sections were washed in TBST, then rinsed with wax, and overlaid with 100 μg mL^-1^ DQ gelatin (Thermo Fisher Scientific, UK) in 50 mM Tris-HCL (pH 7.6), 5 mM CaCl_2_, and 150 mM NaCl for 60 min at 37°C in a humidified chamber. Sections were then washed three times for 5 min in TBST, overlaid with DAPI containing VECTASHIELD, and imaged using fluorescence microscopy.

Reverse transcription-quantitative polymerase chain reactions (RT-qPCR): all explants were snap-frozen in n-hexane, as described above, prior to processing for RNA extraction. Samples were placed in liquid nitrogen-frozen metal chambers containing 0.5 mL of TRIzol reagent (Merck, UK) and homogenised in a dismembrator at 2000 cycles per min for 120 s (Micro-dismembrator U, B. Braun Melsungen AG, Germany); the resulting powder was placed in an Eppendorf tube containing chloroform, vigorously mixed, and microcentrifiuged for 15 min. The clear supernatant was then added to an equal volume of 70% ethanol. The mixture was transferred to a collection column from the RNeasy Plus Kit (QIAGEN, UK), and the supplier’s protocol was followed to isolate total RNA. Complementary DNA was synthesised using 1 μg total RNA and M-MLV reverse transcriptase (Promega, UK) under the supplier’s recommended conditions. QPCR was performed using the GoTaq SYBR Green Master Mix (Promega, UK), 0.3 μM forward and reverse primers, and 12.5 ng of cDNA. Reactions were run on a CFX96 programmable thermal cycler (Bio-Rad, UK) with the following program: at 95°C for 10 min, at 95°C for 30 s, at 60°C for 30 s, and at 72°C for 30 s for 40 cycles. Data are shown as absolute values in nanogrammes as a ratio of 18SrRNA for each sample. Primer sequences were obtained from previously published sources (Khan et al., 2011; Khan et al., 2013; Zhang et al., 2017).

High-density pellet culture: full-depth slices of cartilage were dissected from the metacarpophalangeal joints of immature (7–14-day old) and mature (18–23-month old) bovine steers under sterile conditions. The cartilage slices were temporarily placed in DMEM. Cartilage was finely diced and then incubated for 1 h at 37°C under constant agitation in DMEM high glucose and GlutaMAX™, supplemented with 10 mM HEPES (pH 7.5), 50 μg mL^-1^ gentamicin, and 0.1% (w/v) pronase (from *Streptomyces griseus*, Merck, UK). After the initial incubation, the pronase solution was replaced with 0.06% collagenase (from *Clostridium histolyticum*, Merck, UK) and 2.5% foetal bovine serum, and the cartilage was then digested for 12 h at 37°C under constant agitation. The extract was poured through a Falcon™ 40 μm cell strainer to produce a single-cell isolate. Cells were washed in the chondrogenic medium and re-suspended at a concentration of 0.5 × 10^6^ cells mL^-1^ in the chondrogenic medium, with or without 100 ng mL^-1^ BMP9, in 1.5 mL Eppendorf tubes. The tubes were spun at 500 x *g* for 5 min to generate cell pellets. Medium was replaced every 4th day. Pellets were cultured for 21 days, following which they were fixed in 4% paraformaldehyde in PBS for 12 h at 4°C. The fixed pellets were processed for wax embedding and sectioning.

Statistical analyses: the results are presented as the mean ± standard deviation (s.d.). All datasets involving multiple groups were assessed for normal distribution using the Shapiro–Wilk test and homogeneity of variances using Levene’s test prior to parametric analysis of variance (one-way ANOVA with *post hoc* testing using the Tukey’s method) or non-parametric analysis (the Kruskal–Wallis test with *post hoc* testing using the Conover/Holm method). For analysis of the averages of two groups, we used a two-tailed Student’s t-test. Sample size, replicate descriptions, and details regarding the use of representative datasets are specified in each case in the figure legends. Significant differences are annotated in graphs using asterisks as follows: *p <* 0.05 (*), *p <* 0.01 (**), and *p <* 0.001 (***).

## Data Availability

The raw data supporting the conclusions of this article will be made available by the authors, without undue reservation.
