# Supplementary material for: BMP9 induces postnatal zonal stratification of immature articular cartilage through reconfiguration of the existing collagen framework
Source: Front Cell Dev Biol. 2025 Jan 28;12:1511908. doi: 10.3389/fcell.2024.1511908 (PMC11810917; doi:10.3389/fcell.2024.1511908)
Supplement: Supplementary file 1 [file DataSheet1.pdf]

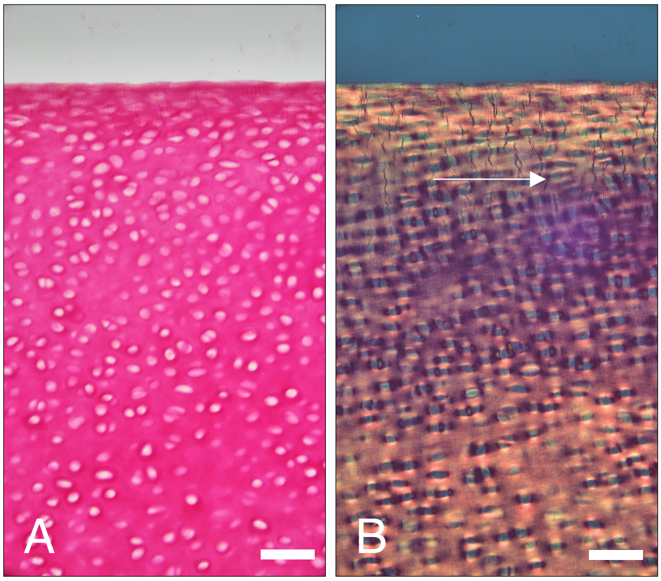

Figure S1. Picrosirius red staining (A) and polarized light microscopy (B) of freshly extracted and fixed immature bovine metacarpophalangeal joint (MCP) articular cartilage. The age of the immature donor was between 1-4 weeks. The direction of collagen fibrils in immature cartilage is predominantly parallel to the surface (*white arrow*) B. Bar = 50μm.

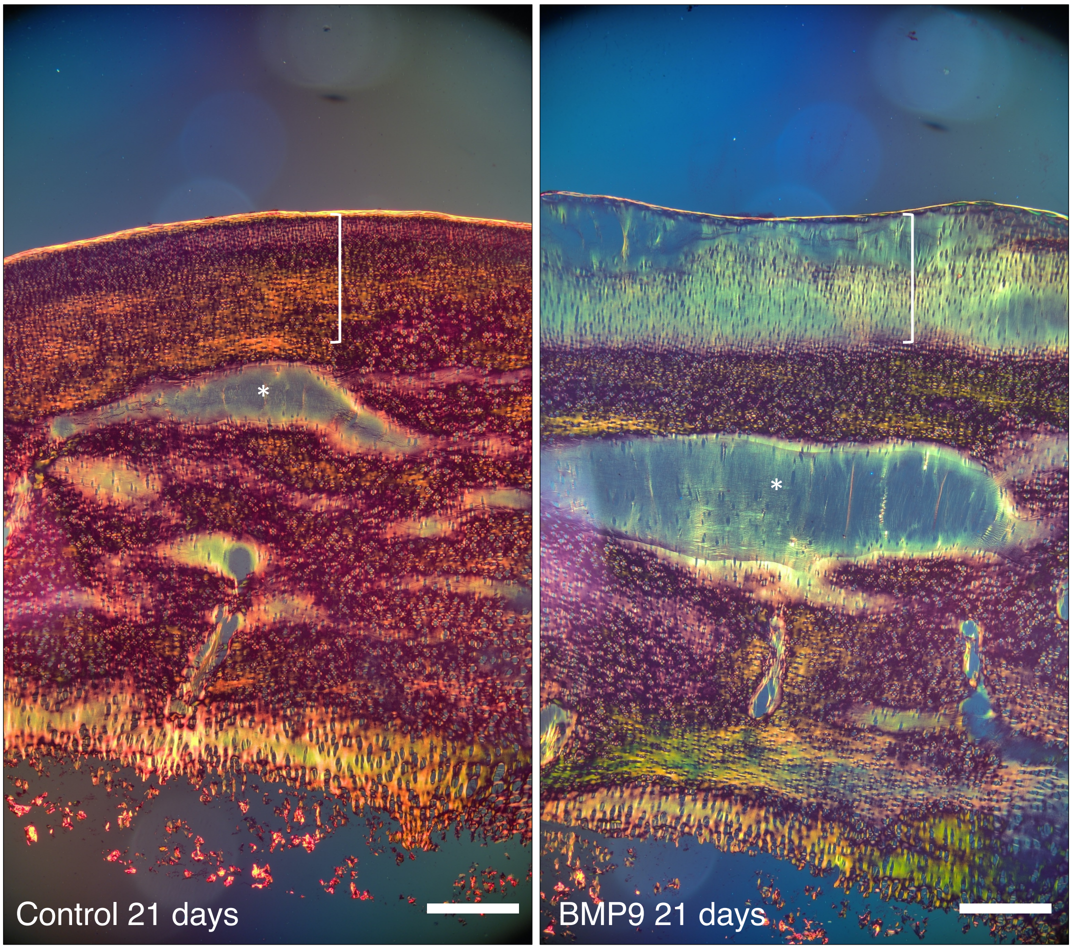

Figure S2. Polarized light microscopy of picrosirius red stained sections of immature bovine articular cartilage treated with BMP9 (100ng mL^{-1}) for 21 days. The images show the surface of BMP9 treated explants undergo profound changes in collagen fibril alignment (*right*) when compared explants grown in the absence of BMP9 (*left*). Immature cartilage is composed of surface permanent cartilage and transient epiphyseal cartilage, and changes in collagen fibril alignment occur in the surface region (variably between 200-700µm below the surface). The inferior epiphyseal region is metabolically active with respect to collagen turnover and it this region which was used as an internal control for anti-COL2¾m labelling and DQ Gelatin assays. The tissue at the centre of both explants is degraded (*asterisk*), this is due to hypoxia and is more colloquially known as the Malteser effect, a common occurrence following long-term culture of cartilage explants. Bar = 500µm.

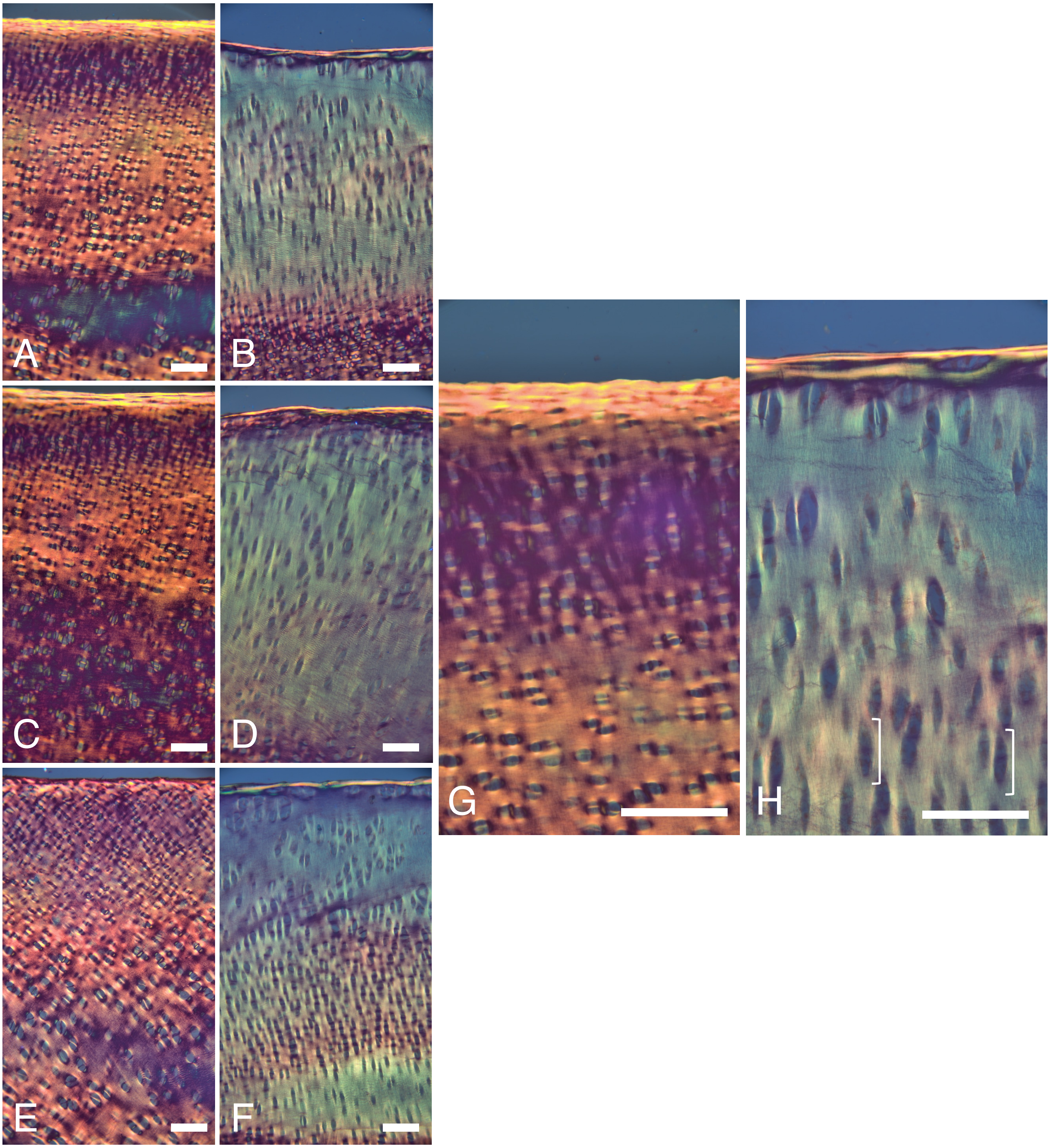

Figure S3. The variability of collagen fibril alignment and cellular morphology of immature bovine articular cartilage explants treated with BMP9. The images show control (**A**, **C**, **E**) and their corresponding BMP9 treated explants (**B**, **D**, **F**) taken from three different donors. Whilst the change in orientation of collagen fibrils is a regular occurrence there is variability between BMP9 treated explants in the depth, cellular density and organisation of these matrices. Bar = 100µm. **G** and **H** are higher-power images of explants shown in **A** and **B**, where the orthogonal arrangement of collagen fibrils, decrease in cellular density, changes in chondron aspect ratio and area in BMP9-treated explants compared to control explants are more obvious (*as quantified in Figure 2*). Elongated chondrons (*bracketed structures*) containing multiple cells are also visible deeper in the tissue. Bar = 100µm
